# Effect of Rare-Earth Ions on the Optical and PL Properties of Novel Borosilicate Glass Developed from Agricultural Waste

**DOI:** 10.3390/ma14195607

**Published:** 2021-09-27

**Authors:** Aiyeshah Alhodaib, Omnia Ibrahim, Suzy Abd El All, Fatthy Ezzeldin

**Affiliations:** 1 Department of Physics, College of Science, Qassim University, Buraydah 51452, Saudi Arabia; Suzy_a_m@yahoo.com; 2 Radiation Chemistry Department, National Center for Radiation Research & Technology, Atomic Energy Authority, Cairo 11787, Egypt; omnia_ibrahim2009@yahoo.com (O.I.); fatthy1uk@yahoo.com (F.E.); 3 Radiation Physics Department, National Center for Radiation Research & Technology, Atomic Energy Authority, Cairo 11787, Egypt

**Keywords:** agriculture waste, neodymium, dysprosium, glass, photoluminescence and optical properties

## Abstract

There is considerable attention devoted to the use of agricultural waste as a raw material substitute for commercial silica in the development of borosilicate glasses doped with rare earth oxides. Here, we present a novel structure for borosilicate glasses made from rice husk ash with a 25% molar ratio of extracted SiO_2_ and doped with neodymium (GRN) or dysprosium (GRD). Adding rare earth oxides to borosilicate glasses by the melt quenching method enhanced optical transmission due to the presence of their tetrahedral geometries. GRN samples showed few bands near zero, which constitutes good utility for band rejection filters in image devices, and the samples exhibited energy values ranging from 3.03 to 3.00 eV before and after gamma irradiation. Optical transmissions of GRD samples showed peaks at 25,974, 22,172, 13,333, 11,273, 9302, 7987, and 6042 cm^−1^. Deterioration in transmittance was observed when the investigated samples were exposed to irradiation doses of 20 and 50 kGy in the wavenumber range of 12,500 to 50,000 cm^−1^; however, different behaviors after irradiation with 50 kGy caused an increase in transparency in comparison to 20 kGy irradiation, which was pronounced for higher wavenumbers (greater than 12,500 cm^−1^). Photoluminescence emission and excitation spectra of the glass-doped Nd^3+^ (GRN) and glass-doped Dy^3+^ (GRD) samples were determined. GRD exhibited emission in the blue and yellow regions of the visible spectrum, which gave a white flash of light. Chromaticity coordinate (CIE) measurements of GRD samples indicated the origin of its luminous color relative to the standard white light region.

## 1. Introduction

There is considerable global interest in agriculture waste management due to increases in waste generated each year, which has a huge mass on land. Solid wastes are difficult to degrade naturally. The most common method for alleviating the solid agriculture waste is burning of these wastes, but this method leaves ash. Scientists have given great attention to completely removing agricultural waste from soil and converting it to valuable products such as glass [1,2]. Glass as a final product from agriculture waste has several benefits depending on the oxides added during the preparation of the glass. Agricultural waste is considered to be a rich source of SiO_2_ and charcoal [3], which serve as raw materials for glass formation. Extracting SiO_2_ from agricultural waste (rice husk) and finding different ways to do so has attracted attention in scientific and commercial fields [4]. Agriculture solid wastes can produce well-known forms of glass as silicates; to enhance the optical properties of the prepared glass [5] (recycled from wastes), rare earth oxides can be added (e.g., Dy_2_O_3_ and Nd_2_O_3_).

Doping of glass with rare earth elements (REs) gives new properties to the glass network and is considered one of the most vital topics in glass science owing to its distinguishing behavior in different applications, especially in the fields of optical communications [6], solid-state lasers (mid-IR laser fabrics) [7], and frequency converter devices [8], which arise from the amazing optical, chemical and physical properties of REs. This glass is also being used in photonic devices, optical fibers, solid-state lasers, wave guides, temperature sensors, and white LEDs [9].

Glass doped with trivalent RE elements has increased hardness and a higher elastic module and chemical durability because of the strength produced in these glass types compared to glass doped with transition metals [10], and they form luminescence glasses with 5d–5d and 4f–4f electronic transitions and exhibit several sharp lines in the UV region and near IR owing to the shielding effects of 5s and 5p orbitals [11,12]. The gap between the ground level and the first excited level is smaller than those of other elements, allowing these glasses to exhibit several bands in their UV spectra [13,14].

Scintillators glasses are transparent glass doped with specific elements (such as dysprosium) that are able to emit white flashes when excited by energetic particles (neutrons, protons, electrons, α-particles, etc.) or photons (γ- or X-rays). They can be used as radiation detectors in numerous fields, such as astrophysics, medical imaging, security devices, nuclear science, geological exploration, and high energy physics [15]. The white flash of light in glasses doped with dysprosium is known to result from emission in two spectroscopic regions, the yellow and blue regions [9].

However, glass-doped neodymium also has been widely applied as a band rejection filter in image display devices; this results from absorption bands arising from transitions in the 4f shell of Nd^3+^ [16,17]. Additionally, Nd^3+^ can produce glass used for halogen lamps, owing to the ability of glass-doped Nd^3+^ to absorb UV rays. Glass-doped Nd^3+^ imparts good chemical durability and high hardness, which makes this type of glass a preferred choice for refractory glasses.

Gamma irradiation can heal or induce defects and lead to formation of color centers inside the glass network; this depends on the rate and type of radiation, the glass composition, or the addition of doping elements. Changes inside the glass network can be followed through the optical, structural, or physical behavior of the glass when exposed to irradiation, and this helps to determine the ability of the glass samples to deal with radiation, which in turn aids in the manufacture of glasses that depress the harmful effect of radiation or other glasses that benefit from the positive effect of radiation.

This work is designed to study the optical transmissions, optical energy band gaps and luminescence properties of borosilicate glasses made from agricultural waste such as rice husk ash and doped with Nd_2_O_3_ and Dy_2_O_3_ to meet quality standards. This demonstration will pave the way for development of new innovative waste composites and for technologically important uses of waste. When doping Nd^3+^ or Dy^3+^ into glass, unique optical properties are obtained. Glass-doped Dy^3+^ can be used in white light applications as scintillators glasses, and it emits light in the yellow and blue regions. Glass-doped Nd^3+^ can be used in band rejection filters in image display devices because it prevents the transmittance of light at several wavelengths due to electronic transitions within its 4f shell. Furthermore, the effect of successive irradiation doses on these properties is discussed.

## 2. Materials and Methods

Glass samples were prepared with a mol% chemical composition of 25% SiO_2_—30% Na_2_O—45% B_2_O_3_, and doped with 4% Dy_2_O_3_ or Nd_2_O_3_ by the conventional technique of melt quenching. The chemical reagents used were high purity Na_2_CO_3_, H_3_BO_4_, Nd_2_O_3_, and Dy_2_O_3_ (Sigma Aldrich, 99.99%, St. Louis, MO, USA).

SiO_2_ was extracted from rice husk. First, rice husk was washed several times with normal and deionized water to remove dust, and then drained of water with a plastic bowl. In the second step, drained samples were leached with HCl (2M hydrochloric acid) for 2 h then drained and washed with distilled water again to remove fabrics. The husk was dried at 120 °C in an oven for 4 h. Finally, the treated rice husk was incinerated at 700 °C for 1/2 h to obtain silica (SiO_2_).

In preparing the glass samples, a digital balance (±0.0001) was used to weigh the molar percentages of the reagents, which were then mixed and stirred for 30 min to obtain a homogenous mixture, preheated at 400 °C for 1 h, and then melted at 1100 °C for 1/2 h. Molten samples were cast on stainless steel plates to form glass samples, which allowed annealing at 400 °C for 1 h. The samples were polished by silicon carbide and cut for measurements.

The structures of the synthesized glass samples were determined via X-ray diffraction (Shimadzu XRD-6000, Nishinokyo, Japan) in the range of 2θ = 4° to 90°. Energy-dispersive X-ray spectroscopy (EDX, Bruker, Mannheim, Germany) was used to verify the elemental compositions of the rice ash and the prepared glass samples by the aiding of mapping method with SEM to give a complete knowledge about homogeneity of the component distribution. Scanning Electron Microscopy (SEM) EM, ZEISS, EVO-MA10, (Jenna, Germany), a V-770 UV-visible/NIR spectrophotometer was used with wavelengths ranging from 190–1800 nm (3200 nm option); for our samples, it was sufficient to measure up to 1900 nm, as no observed peaks were recorded at higher wavelengths. A UV-visible/NIR spectrophotometer was equipped with two detectors; a PMT detector was used to measure the samples in the UV to visible region. A Peltier-cooled PbS detector was used to measure the samples in the NIR region. Photoluminescence spectra were measured at room temperature by a fluorescence spectrometer (Jasco FP-6500, Tokyo, Japan) using a Xenon lamp as the excitation source with a 0.1 s scan speed, 0.25 nm step length and 0.2 nm slit width. Indian Gamma cell (^60^Co) was used as a source of gamma rays with a dose rate of 0.9722 kGy/h at RT.

## 3. Results and Discussion

### 3.1. Structural Identification Technique [XRD, EDX, and SEM]

**Figure 1:**
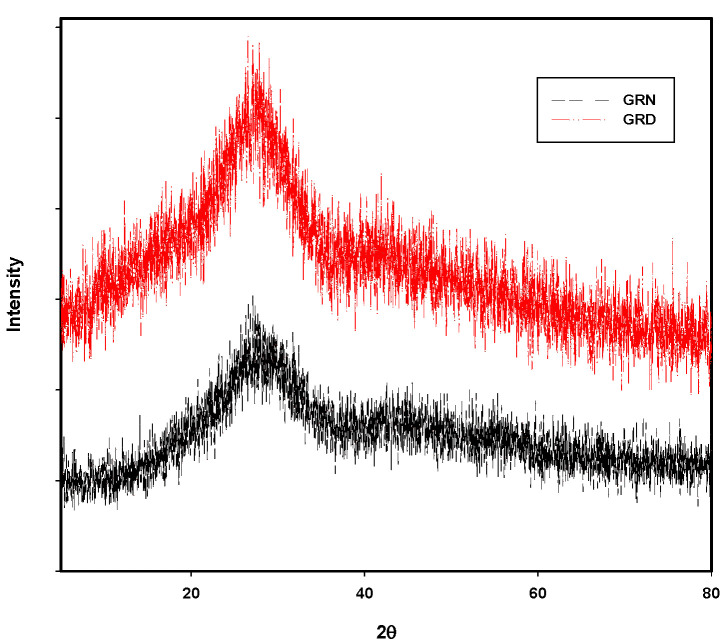
XRD spectra of GRN and GRD glass samples.

The prepared glass samples were checked by X-ray diffraction to determine if the samples were crystalline or non-crystalline in nature. All samples were studied at room temperature. As shown in Figure 1, X-ray diffraction showed a broad band at 2θ = 27 °C that indicated the amorphous nature of the prepared glass samples. EDAX (energy dispersive analysis by X-rays) was carried out to analyze the rice ash components, which showed approximately 87% SiO_2_ yield from treated rice ash.

The same technique was used to determine the average glass composition after preparation. It was found that the element ratios in the glass samples were very near the initial weights of participating elements in the glass preparation, as shown in Table 1.

**Figure 2:**
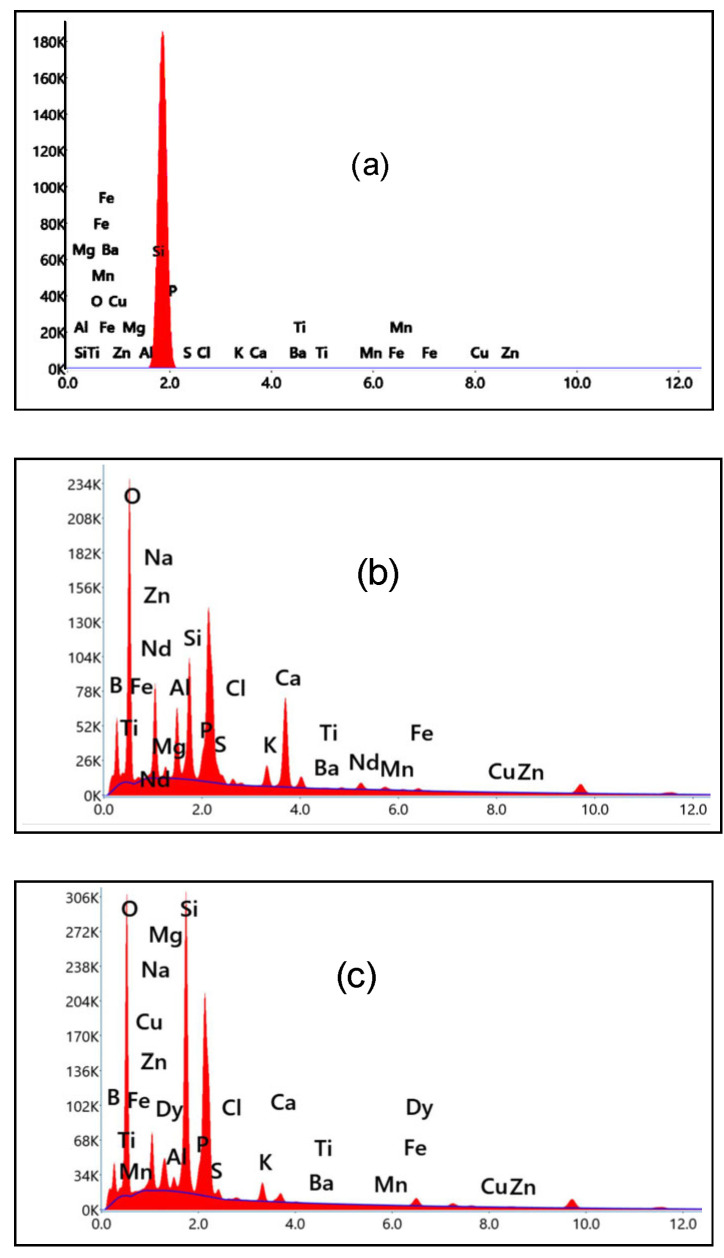
EDX spectra for (**a**) RA, (**b**) GRN, and (**c**) GRD samples.

**Figure 3:**
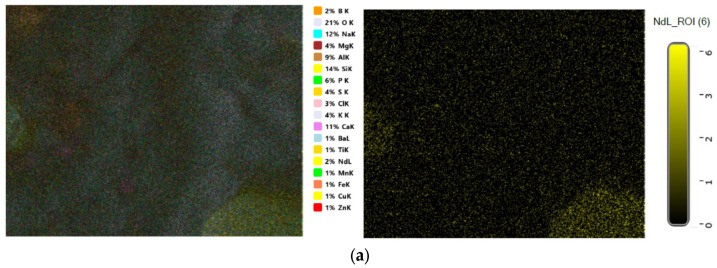

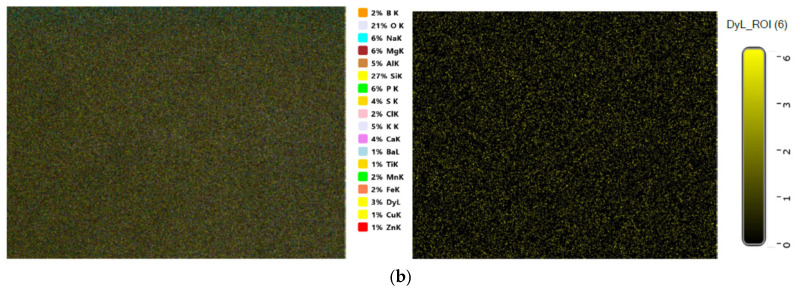
SEM and Elemental mapping for (**a**) GRN (**b**) GRD samples.

EDX spectra of rice ash (RA) (produced by treating and burning rice husk at 700 °C) and the spectra of either Nd^3+^- or Dy^3+^-doped borosilicate glasses (separately extracted from rice ash) in GRN and GRD samples are illustrated in Figure 2, which gives the component percent inside the glass network. Figure 3a,b show images of Scanning Electron Microscope (SEM) provided with elemental mapping spectra for prepared borosilicate glasses doped with Nd_2_O_3_ or Dy_2_O_3_ from rice ash, which illustrates a homogenous distribution of all elements specially doping elements (Nd_2_O_3_ or Dy_2_O_3_) inside the glass matrix.

### 3.2. UV Transmittance Spectroscopy Analysis

#### 3.2.1. Deconvoluted Absorption Spectra of GR Sample

**Figure 4:**
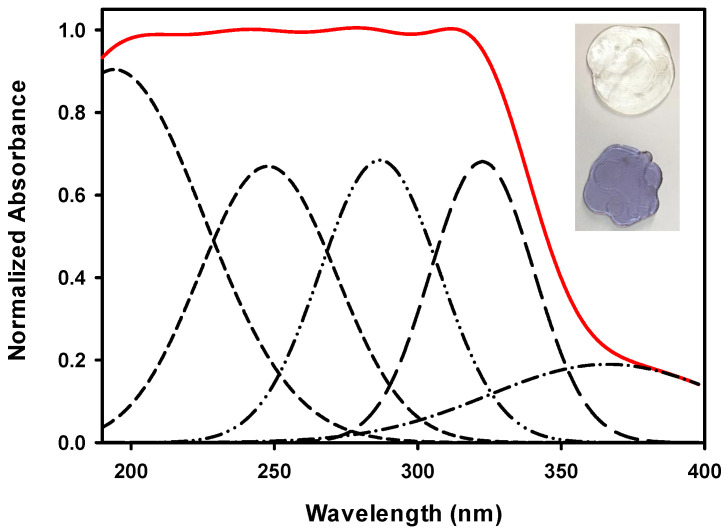
Deconvolution of normalized absorbance spectra of GR with inset pictures of GRN and GRD samples.

The role of doping in the color and transparency of the investigated glasses before irradiation is illustrated in the inset of Figure 4.

**Figure 5:**
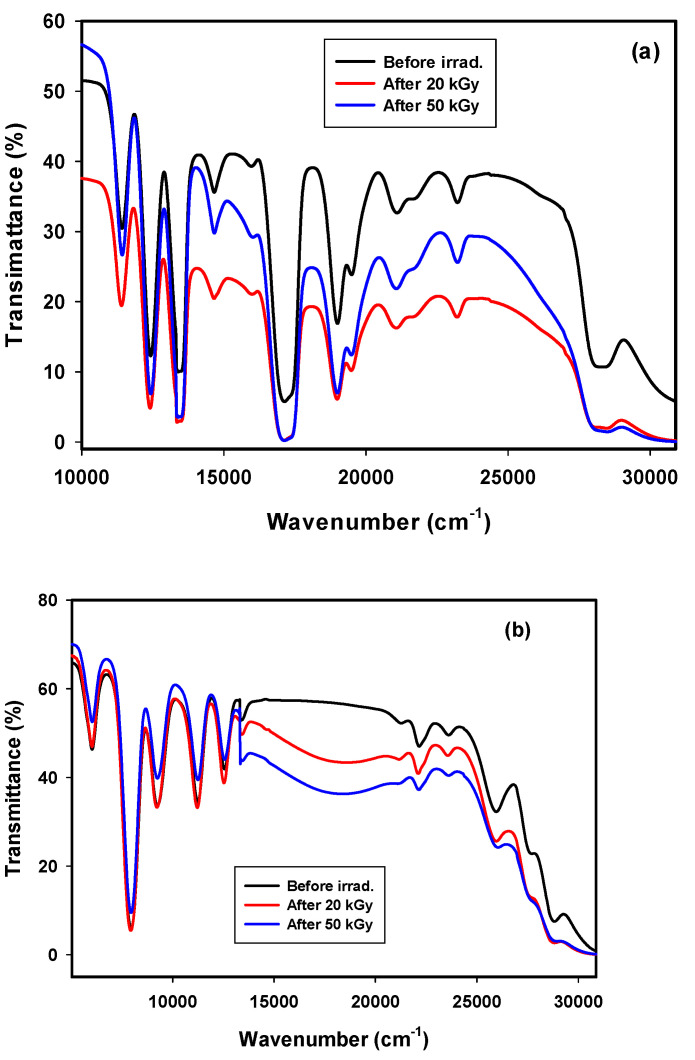
Transmittance spectra of (**a**) GRN and (**b**) GRD samples at room temperature.

GRN samples showed a transparent purple color, and GRD had a colorless transparent appearance. This figure depicts the optical absorption spectra of undoped borosilicate glass extracted from rice husk (GR) in the wavelength range 200 to 1800 nm. The spectrum of the GR glass revealed a broad UV-Vis absorption band extending from 190 to 400 nm. The spectrum showed only distinct and broad UV absorption in the UV region up to 370 nm. After deconvolution of the spectrum, five peaks at 197, 245, 285, 322, and 365 nm could be identified for the undoped glass, which is expected to be related to two types of defects. The first is for silicon-electron-center defects (Si-ECs), and the other is for silicon-oxygen-hole-center defects (SiOHCs) at 245 and 285 nm [18,19]. Another absorption peak at 365 nm is associated with Fe^3+^ ions [17], while peaks at 197 nm are attributed to the absence of one or more electrons from Non Bridging Oxygen’s (NBOs) to give Non Bridging Oxygen’s Hole Centers (NBO-HCs) in addition to the creation of HCs of Al inside the glass system from rice ash [18,20]. Peaks near 322 nm can appear due to the presence of borate groups. However, the UV-visible-NIR region showed 9 distinct bands for the GRN glass and 8 distinct bands for the GRD glass, as shown in Figure 5a,b.

#### 3.2.2. Transmittance Spectra of GRN

Transmittance spectra of borosilicate glass extracted from rice husk and doped with 4% neodymium oxide (GRN) before and after irradiation with 20 and 50 kGy gamma rays are shown in Figure 5a. In general, Nd^3+^ showed different behaviors in amorphous fields and crystalline fields. In an amorphous field, the transition of an excited electron occurs between the ground and excited levels, where each band consists of multiple random levels resulting in homogenous broadening in the crystalline host, where Nd^3+^ can be located in a regular crystal field [21]. Several bands were observed in the spectra because of the electron transitions from the ground level (^4^I_9/2_) to higher energy levels in the 4P^3^ configuration of Nd^3+^, [22]. The spectrum reveals small UV transmittance bands at 28,409, 23,255, 21,141, and 19,531 cm^−1^ for transitions to higher levels (^4^I_11/2_ + ^4^D_3/2_ + ^4^D_5/2_), ^3^P_1/2_, (^4^G_11/2_ + ^2^K_15/2_ + ^2^G_9/2_ + ^2^D_3/2_), and (^4^G_9/2_+^2^F_13/2_+^4^G_7/2_), respectively. Then, stronger bands at 19047, 17,182 cm^−1^ were related to other transitions to (^4^G_5/2_ + ^2^G_7/2_) and ^2^H_11/2_ states, and a weak band at 14,684 cm^−1^ indicated the transition to the ^4^F_9/2_ level; finally, three medium bands at 13,422, 12,437, and 11,415 cm^−1^ were related to (^4^S_3/2_ + ^4^F_7/2_), (^4^F_5/2_ + ^2^H_9/2_), and ^4^F_3/2_ transitions, respectively. Other bands at 30,769 cm^−1^ were related to Fe^3+^ impurities in the raw materials; the 27,027 cm^−1^ band was related to the structure of BO_3_ and NBO-HC from silica, while the band at 15,923 cm^−1^ was related to Fe^2+^. The band at 17,241 cm^−1^, which was assigned to the transition ^4^I_9/2_ → ^4^G_5/2,_ is the most hypersensitive band depending on the surrounding environment of Nd. This band follows the selection rules |ΔJ| ≤ 2, |ΔL| ≤ 2 and |ΔS| = 0 [23]. Generally, the band at 12437 cm^−1^ is used to pump Nd^3+^ activated lasers with semiconductor laser diodes (GaAs) [24]. The GRN glass sample shows several bands at 52,631 → 30,769, 28,409, 19,047, 17,241, 13,440, and 12,391 cm^−1^, with transmittance values near 0%; the transmittance of these wavelengths can be prevented by this type of glass, so this glass is recommended for use as an effective band rejection filter. The transmittance values of these peaks move closer to zero after irradiation with 20 and 50 kGy.

Gamma rays are ionizing radiation that induces several new properties in glass due to the creation of new defects, and they can also generate new color centers by capturing excited electrons and forming positive holes. When the GRN sample was exposed to 20 and 50 kGy gamma rays, the optical transmittance spectra were similar, but bands had lower transmittance due to the capture of electrons produced by gamma irradiation by Nd^3+^ ions. No additional bands appeared after irradiation. Gamma irradiation plays an effective role in enhancing the function of the prepared glass as a band rejection filter, since after irradiation the intensities of the observed peaks approached zero % transmittance.

The indirect energy gap (E_g_) for glass-doped Nd was observed to decrease from 3.03 to 3.022 and then to 3.00 eV after irradiation with 20 and 50 kGy doses, which is dominant in the sample as it is glass-amorphous in nature. While a small amount of the component of the glass matrix may seem to distribute inside the glassy network by crystalline order giving direct energy gap values as following 3.52, 3.49, 3.42 eV before and after irradiated with 20 and 50 kGy; respectively. The energy gap determines the energy of absorbed light; this decrease in E_g_ values is related to an increase in the number of NBOs produced, indicating that the energy required to absorb light decreases. The direct and indirect optical energy gaps can be calculated as below, in which α represents the absorption coefficient, υ is the frequency, and B is a constant:

α(υ) = B(hυ − E_g_)^n/^hυ (1)

**Figure 6:**
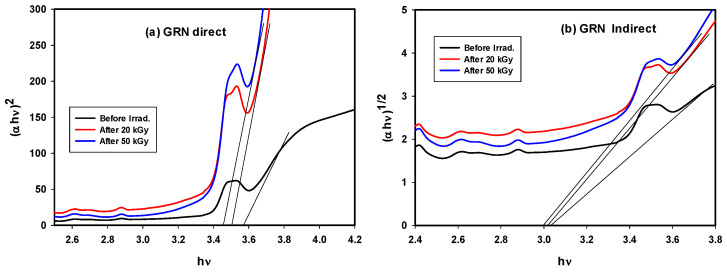
Tauc plot of GRN samples before and after gamma irradiation doses for (**a**) direct and (**b**) indirect energy gap.

This gives a linear relation between [(αhυ)^1/2^ or (αhυ)^2^] and αυ, and E_g_ values may be obtained from linear extrapolation to zero, as shown in Figure 6a,b and Table 2.

#### 3.2.3. Transmittance Spectra of GRD

Figure 5b shows transmittance spectra for the GRD glass before and after irradiation. Bands were observed in relatively similar locations, but with remarkable changes in intensities. No additional bands appeared after irradiation. Several bands were observed at 25,974, 22,172, 13,333, 11,273, 9302, 7987, and 6042 cm^−1^; these were derived from Dy^3+^ ions as a result of broadening of the f-f transition resulting from the ^6^H_15/2_ ground state into (^4^I_13/2_ + ^4^F_7/2_), ^4^I_15/2_, ^6^F_3/2_, ^6^F_7/2_, (^6^H_7/2_ + ^6^F_9/2_), (^6^F_11/2_ + ^6^H_9/2_), ^6^H_11/2_ excited states, respectively [25,26,27]. These bands were broad due to the amorphous nature of the glass system [28]. However, the band at 7987 cm^−1^ is an intense band that obeys the selection rules |Δ S| = 0, |Δ L| ≤ 2, and |Δ J| ≤ 2 [29], and is known as a hypersensitive transition that is due to the transition ^6^H_15/2_ → ^6^F_11/2_ + ^6^H_9/2_.

After irradiation with 20 and 50 kGy doses, the intensities of the peaks change along with wavelengths. From spectra beginning at 12,500 cm^−1^, the transmittance decreased after gamma irradiation, especially at 17,241 cm^−1^; this was due to an effect induced by borate as a result of NBO conversion of BO_3_ into BO_4_ groups, which overlapped with NBO hole centers in silica after irradiation. Additionally, the presence of impurities such Fe^3+^ in rice husks and other chemical raw materials played an obvious role in the changes in this area due to the conversion of Fe^3+^ to Fe^2+^ under the influence of radiation. The transmittance of the GRD sample increased after irradiation in the visible region and was more pronounced in the NIR region due to the capture of electrons produced by gamma irradiation involving Dy^3+^ ⟷ Dy^3+^ ions. The observed high transparency in the visible and NIR regions revealed that the composition possessed a promising scintillation character, which was confirmed by the following photoluminescence studies.

**Figure 7:**
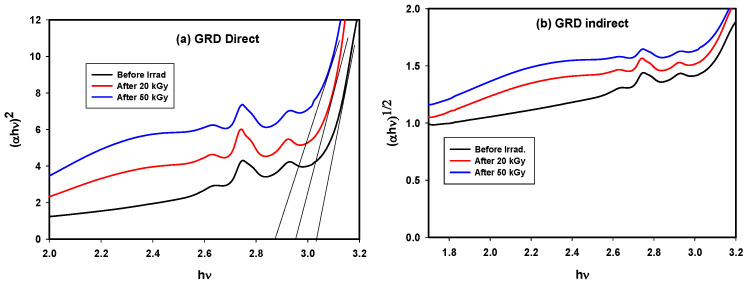
Tauc plot of GRD samples before and after gamma irradiation doses for (**a**) direct and (**b**) indirect energy gap.

Lowering of indirect E_g_ values from 2.90 to 2.81 and then to 2.78 eV also lowering in direct E_g_ from 3.03 to 2.97 and then 2.87 eV was observed after irradiation due to increases in NBOs and defects as illustrated in Figure 7a,b and Table 2, where the Dy^3+^ ions present in the glass structure are unable to absorb all of the excited electrons ejected by irradiation. The change in the energy gap after gamma irradiation was evidence that modifications occurred due to the effects of radiation.

### 3.3. Photoluminescence

#### 3.3.1. Photoluminescence of GRN

Neodymium is a lanthanide metal that has a [Xe] 4f^4^ 6s^2^ electronic configuration in the ground state. Excitation of electrons in the 4f shell allows transitions that result in emission after irradiation at 585 nm, while electrons in other orbitals do not contribute to luminescence. The photoluminescence of Nd^3+^ can be ascribed to the transition between the excited level 4f^2^ 5d^1^ and ground level 4f^3^, and the resulting emission depends mainly on the structure of the host material [30,31].

**Figure 8:**
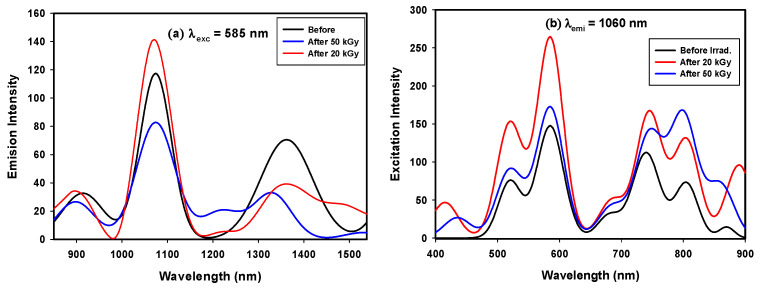
**(a)** Emission spectra and (**b**) Excitation spectra of GRN samples before and after gamma irradiated.

Emission and excitation spectra resulting from excitation at λ_exc_ = 585 nm and emission at λ_emi_ = 1060 nm are shown in Figure 8a,b. Figure 8a shows 3 emission peaks at 926, 1072, and 1353 nm. Borosilicate glass-doped Nd ions were pumped with an output laser at 585 nm that gave resonance with the ^4^F_5/2_ + ^2^H_9/2_ level of neodymium and excited the ^4^F_3/2_ metastable level, giving broad emissions at 926 nm for ^4^I_9/2_, 1072 nm for ^4^I_11/2_, and 1353 nm for ^4^I_13/2_. This broad emission was due to the amorphous nature of the host. The transition ^4^F_3/2_ → ^4^I_11/2_ showed the highest peak intensity due to the lasing transition power of this glass [32,33]. The expected reason for the decreased intensities of the other two peaks compared to that for ^4^I_11/2_ involves the capability of Nd^3+^ ions to accept excited electrons in the ground state. After irradiation, the intensity of the ^4^F_3/2_ → 1072 nm transition increased after a 20 kGy dose and then decreased slightly with a 50 kGy dose, which can be explained by the quenching effect of Nd^3+^. Figure 8b shows six excitation peaks at 515, 595, 690, 730, 812, and 880 nm; by increasing the γ-ray dose, the excited Nd^3+^ ions form a cluster of Nd^3+^ and this promotes multiphonon relaxation and subsequent quenching [16].

#### 3.3.2. Photoluminescence of GRD

**Figure 9:**
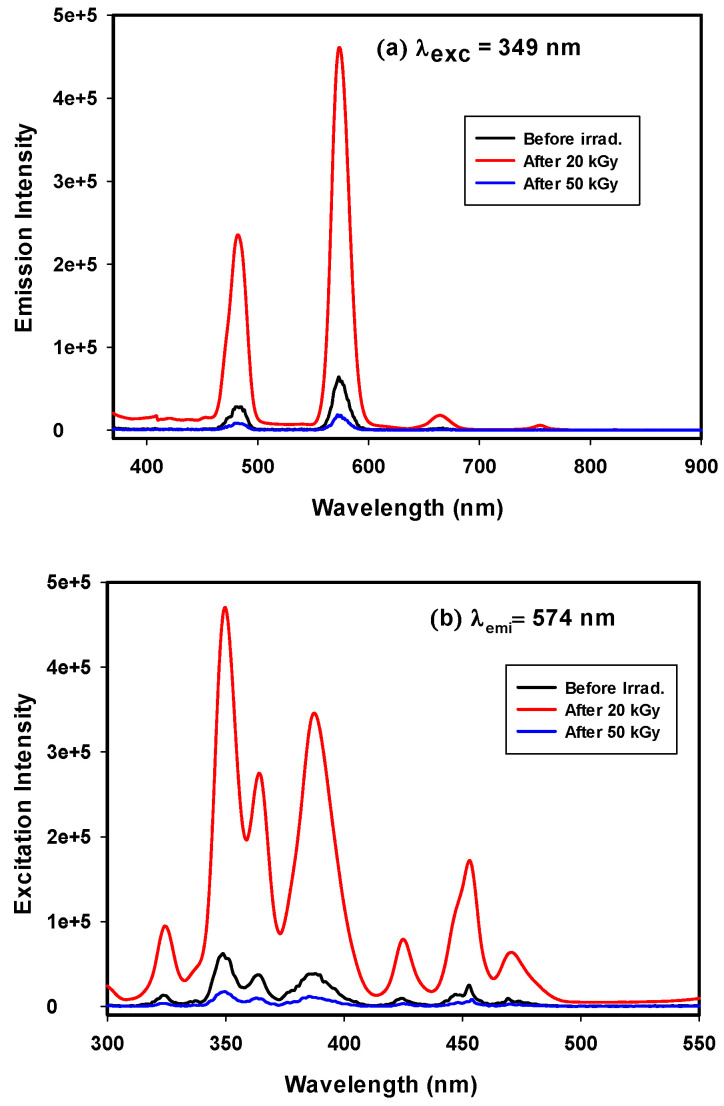
(**a**) Emission spectra and (**b**) Excitation spectra of GRN samples before and after gamma irradiated.

Dy^3+^ is one of the important candidate rare earth elements to be applied for white-light applications, due to its emission in the yellow and blue regions [9]. Therefore, if glass doped with Dy^3+^ is excited in the UV/blue region, it emits white light to be used for W-LEDs. Photoluminescence excitation for borosilicate glass doped with Dy^3+^ can be detected with an emission wavelength of 574 nm, as shown in Figure 9a. Figure 9a shows an excitation spectrum with 8 peaks at 323, 337, 347, 363, 386, 423, 451, and 473 nm, resulting from f-f electronic transitions from ^6^H_15/2_ to (^4^M_17/2_ + ^6^P_3/2_), ^4^I_9/2_, ^6^P_7/2_, (^4^I_11/2_ + ^6^P_5/2_), (^4^I_13/2_ + ^4^F_7/2_), ^4^G_11/2_, ^4^I_15/2_, and ^4^F_9/2_, respectively [34].

Photoluminescence emission spectra containing 4 emission bands for borosilicate glass doped with Dy^3+^ ions are shown in Figure 9b, with λ_exc_ = 349 nm. Two small bands are seen at 665 and 754 nm, two intense bands are seen at 482 nm in the blue region for the ^4^F_9/2_ → ^6^H_15/2_ transition owing to the effect of the magnetic dipole transition, and another is seen at 570 nm in the yellow region for the ^4^F_9/2_ → ^6^H_13/2_ transition arising from the hypersensitive transition from the forced electric-dipole that obeys the selection rule |ΔS| = 0, |ΔL| ≤ 2 and |ΔJ| ≤ 2. Moreover, the intensity of the peak at 482 nm for the ^4^F_9/2_ → ^6^H_15/2_ transition is lower than the intensity at 570 nm of the ^4^F_9/2_ → ^6^H_13/2_ transition. It is known that ^4^F_9/2_ → ^6^H_13/2_ is a hypersensitive transition that depends mainly on the host matrix; on the other hand, ^4^F_9/2_ → ^6^H_15/2_ results from the strength of the crystal field in the matrix. The dominant appearance of the ^6^H_13/2_ transition ensures the symmetric distribution of Dy^3+^ in the glass matrix environment [35].

The emission band shapes and distributions were identical before and after irradiation, which indicated the shielding of the 4f shell by the electrical field originating from the outer 5s^2^ 5p^6^ electrons. The only intensity changes occurred due to self-emission inside the glass network under radiation, which is known as the quenching effect. The quenching effect occurred due to interactions between gamma rays and the glass matrix that were different from those of the UV excitation process. In UV excitation, there was direct excitation of luminescent ions (Dy^3+^), but gamma rays react with defects (holes, color centers, electrons) in the glass matrix to produce extra excited electrons that transfer directly or indirectly to conductive levels. The observable white light flash is due to the effect of X-rays, which is similar to the gamma ray effect occurring in a dark chamber; due to the difficulty of following the gamma reaction on glass by eye, there is an opaque cover of the chamber. This allows use of the prepared glass-doped Dy^3+^ as a gamma or X-ray detector for safety detector glass in reactors and laboratories.

White luminescence is generated by mixing blue, green, and red colors produced by the appropriate ratio of three primary red, green, and blue emissions or from mixing yellow and blue emissions [36]. In the case of white luminescence generated from yellow and blue mixtures, the Y/B ratio must be calculated to determine the local symmetry of the doping luminescent material inside the environment and the electronegativity surrounding the rare earth ions [37]. In Dy^3+^-doped borosilicate glasses extracted from rice ash, the ratio between the intensities of yellow emission and blue emission (Y/B ratio) is linked to ^4^F_9/2_ → ^6^H_13/2_ and ^4^F_9/2_ → ^6^H_15/2_ transitions [38]. The asymmetric nature of the prepared glass is shown to increase, as indicated by an increase in the intensity of the ^4^F_9/2_ → ^6^H_13/2_ electrical dipole transition [39]. The current work gives Y/B ratios of 2.269 and 2.115 for Dy^3+^-doped borosilicate glass before and after irradiation with a 50 kGy dose, respectively, as given in Table 3. Y/B ratios provide information on the ability of the studied samples to generate white or yellow light, which differ according to the glass-network environment or Dy^3+^ concentration. The increase in the Y/B ratio points to the degree of covalent bonding “Dy^3+^-O^2-^” in the higher local symmetry of the dysprosium environment [40].

1. Decay curve for GRD samples

**Figure 10:**
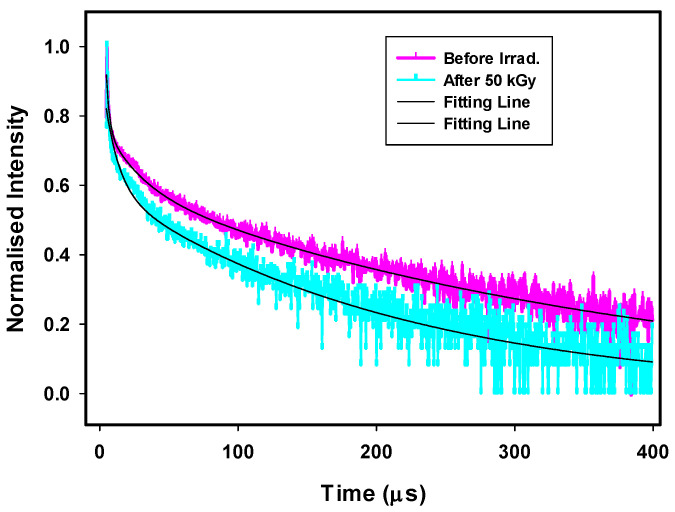
Luminescence decay profiles of the 4F9/2 level of GRD glasses before and after gamma irradiation.

Decay lifetimes were measured with λ_exc_ = 350 nm for the borosilicate glasses doped with Dy^3+^ (GRD) before and after gamma irradiation, as shown in Figure 10. The decay profile was evaluated to follow emission at 482 nm from the ^4^F_9/2_ excited state level. It was fitted with exponential equations giving information about the interactions occurring between Dy^3+^-Dy^3+^ ions, in which one of them is the donor (excited) and the other is the acceptor (unexcited) [41].

The curves exhibited three components related to fast, medium, and slow relaxation of ions processes that are expressed by τ1, τ2, and τ3 with values 0.70, 15.34, and 89.33 μs with contribution percentage about 6480, 822, and 203; respectively for unirradiated GRD glass sample, while after irradiated with 50 kGy, decay processes (τ1, τ2, τ3) are equal to 0.65, 14.41, and 76.95 μs with contribution percentage 3518, 335, and 73, respectively. The observed three lifetime quenching process can describe the life time needed for the excited electron in the excitation transition level before its movement to the ground transition level. The energy transfer mechanism can be followed from (^4^F_9/2_) excited energy level of Dy^3+^ ion to the nearest other Dy^3+^ in its ground level (^6^H1_5/2_). This process makes the first Dy^3+^ ion in intermediate level (^6^H_9/2_), while the second Dy^3+^ ion becomes ^6^F_3/2_ that is in resonance with ^4^F_9/2_ → ^6^H_9/2_ transition. Finally, both Dy^3+^ ions give fast non-radiatively decay to the ground state. The distance between Dy^3+^ ions is the main effective parameter in this mechanism. Therefore, by increment created ions concentration, ion–ion space decreases and the interaction increases. Upon being irradiated the sample, Dy^3+^ ions liberated and increased in the matrix, leading to a decrease in decay lifetime. The average decay times can be expressed with the following formula:

τ_avg_ = (A_1_τ_1_^2^ + A_2_τ_2_^2^ + A_3_τ_3_^2^)/(A_1_τ_1_ + A_2_τ_2_ + A_3_τ_3_) (2)

where A_1_, A_2_, and A_3_ are fitting constants as weight coefficient and τ_1_, τ_2_, τ_3_ are the decay times in μs. τ_avg_ is approximately 515.4338 μs and decreases to 395.822 μs after a 50 kGy irradiation dose, indicating the existence of ET (Energy Transfer) between the acceptor and donor [40,41].

2. CIE color chromaticity co-ordinates

**Figure 11:**
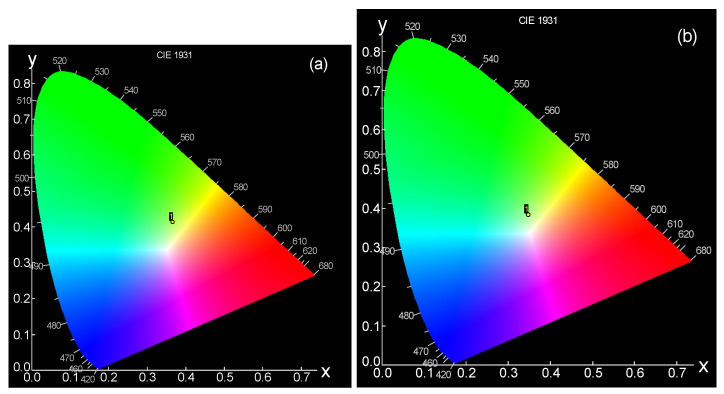
CIE coordinate diagram of (**a**) GRD before irradiation and (**b**) GRD after irradiated with 50 kGy.

Any object can reflect or produce light that causes color, and every color has a special point on the chromaticity diagram. A chromaticity diagram, which is named a color map, can be used to indicate the color coordinates of the material with a specified point on the diagram [36]. A chromaticity coordination can be calculated according to the “Commission International de I’Eclairage 1931 (CIE)” to determine the performance of Dy^3+^-doped borosilicate glasses. Color coordinates are a very important parameter for determining the ability of luminescent glasses to be used in white LED applications. According to emission spectra, CIE color coordinates were obtained for the studied samples. Borosilicate glass-doped Dy^3+^ before and after irradiation with a 50 kGy dose gave CIE color coordinates (x, y) of (0.37, 0.415) and (0.365, 0.398), respectively, as given by Table 3; this can be depicted with the chromaticity diagram (CIE 1931) shown in Figure 11a,b for samples before and after 50 kGy irradiation. The CIE color coordinates were located very near the white region upon irradiation and moved closer to the white region, giving a white flash with higher power.

In addition, white color quality can be determined as the correlated color temperature (CCT) from the equation proposed by McCamy [42] as follows:

CCT = −449n^3^ + 352n^2^ − 682n + 5520.33 (3)

where the epicenter can be expressed as (xe = 0.332, ye = 0.186) and n is equal to (x–xe) divided by (y–ye). CCT can determine the nature of the resulting light and whether it appears as “warm’’ or ‘‘cool’’. It is known that “warm” light has a CCT value less than 3200 K and “cool” light has a CCT value greater than 4000 K [42]. Dy^3+^-doped borosilicate glasses before and after irradiation gave CCT values above 4000 K, as tabulated in Table 3, which confirms the ability of these glass types to emit white light with a cool nature; the sample exhibited a CCT value of 4483.09 before irradiation and this increased to 4541.93 after irradiation.

## 4. Conclusions

In summary, we have developed a novel glass system by inserting rice husk ash components and using the melt-quenching technique with two different systems to be used for varied applications. Not only has this allowed for beneficial employment of agriculture wastes, but surprisingly, the GRN samples exhibit potential for application as band rejection filters at different wavelengths and the GRD samples emit in the yellow and blue regions and give a white flash; this confirms the utility of these glasses as scintillators glass. The unique structure also results in improved transmittance and photoluminescence properties, which simplifies many aspects of fabrication and expands the opportunities for several optical devices. Although the doped borosilicate glasses exhibited more than one transmittance band due to electronic transitions in both samples, GRN samples gave several for the glass network and the doped Nd^3+^ ions. The GRN glass sample gave more than one transmittance band with intensity near 0% due to inter-transitions in its 4f shell, which allows use of this sample as a band rejection filter at the different wavenumbers. However, in the transmittance spectra of the GRD samples, several bands are observed related to Dy^3+^ ions transitions from the ^6^H_15/2_ ground state to the (^4^I_13/2_ + ^4^F_7/2_), ^4^I_15/2_, ^6^F_3/2_, ^6^F_7/2_, (^6^H_7/2_ + ^6^F_9/2_), (^6^F_11/2_ + ^6^H_9/2_), and ^6^H_11/2_ excited states, respectively. For GRD samples, deterioration in the transmittance was observed when the investigated samples were exposed to irradiation doses of 20 and 50 kGy in the wavenumber range of 12,500 to 50,000 cm^−1^; however, irradiation with a 50 kGy dose caused a greater increase in transparency than a 20 kGy dose, and the difference was more pronounced for higher wavelengths. PL measurements of GRN samples with λ_exc_ = 585 nm gave three emission peaks at 926, 1072, and 1353 nm for the transition from ^4^F_3/2_ to ^4^I_9/2_, ^4^I_11/2_, and ^4^I_13/2_, respectively, indicating the homogenous distribution of Nd^3+^ ions in the borosilicate matrix. GRD samples emitted in the yellow and blue regions with λ_exc_ = 349 nm, which resulted in a white flash, confirming the utility of these glass types as scintillators glass. The emitted white light had a cool nature, as the CCT values of the samples were above 4000 K. Additionally, the Y/B ratio was calculated as 2.269 and 2.115, values that confirmed the symmetrical distribution of Dy^3+^ ions in the surrounding media. This is the first report of borosilicate glasses doped with Nd^3+^ or Dy^3+^ and is made from borosilicate extracted from rice ash waste. We believe that this represents a major step towards production of high-performance glass that can be integrated with other glass systems derived from agricultural waste.

## Figures and Tables

**Table 1:** EDX Analysis of RA, GRN, and GRD Samples.

Sample	Elements																		
	O	Na	Mg	Al	Si	P	S	Cl	K	Ca	Ti	Mn	Fe	Cu	Zn	Ba	B	Nd	Dy
RA	0.14	0.5	0.4	0.4	87.0	6.5	0.01	0.05	0.19	0.04	0.01	0.32	1.89	0.43	0.49	0.13	1.5	0	0
GRN	36.2	6.87	0.67	0.9	21.01	5.02	4.93	0.43	2.35	1.9	0.02	0.11	1.99	0.33	0.82	0.35	11.5	4.59	0
GRD	34.4	5.83	1.05	0.83	18.55	5.13	5.53	0.14	2.58	1.46	0.03	0.13	1.34	0.31	0.86	0.13	12.6	0	9.09

**Table 2:** Energy Gap Values for GRN and GRD Glasses Before and After Gamma Irradiated.

Sample	Direct Energy Gap			Indirect Energy Gap		
	Before Irrad.	After Irrad.		Before Irrad.	After Irrad.	
		20 kGy	50 kGy		20 kGy	50 kGy
**GRN**	3.52	3.49	3.42	3.03	3.022	3.00
**GRD**	3.03	2.97	2.87	2.90	2.81	2.78

**Table 3:** Yellow to blue integrated intensity ratios, average decay time, chromaticity color coordinates, and CCT of GRD glasses before and after irradiation.

Sample	Y/b	Color Co-Ordination (x,y)		CCT
		X	Y	
Before Irrad.	2.269	0.370	0.415	4483.09
After 20 kGy	1.958	0.375	0.377	4157.73
After 50 kGy	2.115	0.365	0.398	4541.93

## Data Availability

The data presented in this study are available on request from the corresponding author.
